# Supplementary material for: The Effects of an 8-Week Cognitive–Motor Training Program on Proprioception and Postural Control Under Single and Dual Task in Older Adults: A Randomized Clinical Trial
Source: Healthcare (Basel). 2024 Nov 17;12(22):2297. doi: 10.3390/healthcare12222297 (PMC11594176; doi:10.3390/healthcare12222297)
Supplement: Supplementary file 1 [file healthcare-12-02297-s001.zip › healthcare-3083945-supplementary.pdf]

S1: sessions designed

FIRST SESSION: individual

Music: sube la mano pa arriba (3min28s); saltando King Africa (3min37); Echame la culpa Luis Fonsi (3min30s); Una lluna al aigua (4min27s); Despacito (4min42s); Qué bonito es querer (4min34s).

First Section:

1. **First beat:**

a. In four counts, walk forward; in four counts, stand on tiptoes.

b. In four counts, walk backward; in four counts, stand on one leg.

c. Same as the first.

d. Same as the second but with the opposite leg.

2. Second baat:

a. Four counts of lateral step touch, four counts of a quarter squat.

b. Four counts of step touch returning to the starting point, four counts of a quarter squat.

c. Same going forward.

d. Same going backward.

3. Third beat:

a. Eight counts for a four-pointed star (forward, side, back, opposite side).

b. Same, returning to the starting point.

c. Four-pointed star with the opposite foot.

d. Same, returning to the starting point.

4. Fourth beat:

a. Twists to the right.

b. Twist forward.

c. Twist to the left.

d. Twist backward.

5. Fifth beat:

a. Rock and roll with the right leg lifted to the right.

b. Same to the left.

c. Same while making a circle to the right.

d. Same while turning to the left.

Second Section: Pairs

1. <u>First beat:</u>

e. Holding hands with your partner, three lateral steps + lift one knee.

f. Same to the other side.

g. Forward + lift both knees.

h. Backward + lift both knees.

<u>2. Second baat:</u>

i. Squats facing your partner, moving up and down.

j. Same but looking forward.

k. Same with your backs against each other.

l. Same with your back to the instructor.

<u>3. Third beat:</u>

m. Balance while leaning forward with feet grounded, holding your partner's weight.

n. Same with the other partner.

o. Same while standing on one leg (dominant leg).

p. Same with the other partner.

<u>4. Fourth beat:</u>

q. Shoulder-to-shoulder balance, supporting your partner's weight.

r. Same with the other partner.

s. Same while lifting the leg furthest from your partner.

t. Same with the other partner.

<u>5. Fifth beat:</u>

u. Dance intertwined by the hands.

v. Same, spinning on one leg.

w. Touch your partner’s feet.

x. Dance intertwined by the hands again.

Third Section: Trios

Music: "Vivir Mi Vida" by Marc Anthony (4 min); "Music de Carrer" by Txarango (4 min 19 s); "Marcha, Marcha" (4 min 56 s); "Bésame" by David Bisbal (3 min 26 s); "Bailando Por Ahí" by Juan Magan (3 min); "Lloverá y Lloveré" by La Pegatina (2 min 57 s).

1. <u>First beat:</u>

y. Conga, alternating leg raises with hands on the person in front.

z. Same with one arm up and the other resting on your partner.

aa. Same with the opposite arm.

bb. Conga with three kicks from each leg.

<u>2. Second baat:</u>

cc. Holding hands with arms extended in a triangle, do squats.

dd. Same on one leg.

ee. Same with the other leg.

ff. One hand in the center (star position), changing every four counts.

<u>3. Third beat:</u>

gg. Squats with backs pressed together.

hh. Same, but when you reach the bottom, lift one leg.

ii. Same with the other leg.

jj. Squat, and at the bottom, push with your backs.

<u>4. Fourth beat:</u>

kk. Balance while holding hands in a triangle, extending one leg backward.

ll. Hold for a count of seven, then return to the start.

mm. Same with the other leg.

nn. Hold with the other leg and return.

5. Fifth beat:

oo. Dance while tapping feet in the center without holding hands.

pp. Same.

qq. Tap feet with the partners next to you, alternating feet.

rr. Same.

Fourth Section: Groups of Four

1. First beat:

ss. Sardana dance to one side, all holding hands.

tt. Hold with knees raised for a count of eight, arms up.

uu. Sardana dance to the other side.

vv. Hold with knees raised.

2. Second baat:

ww. Sardana with a quarter squat in four counts.

xx. Same to the other side.

yy. Sardana again with a squat on one leg.

zz. Same to the other side with the opposite leg in the squat.

3. Third beat:

aaa. Line formation, grabbing the opposite leg with the hand on the shoulder.

bbb. Same.

ccc. Change direction and grab the opposite leg.

ddd. Same.

4. Fourth beat:

eee. Twists in a circle while holding hands, moving around.

fff. Same in the opposite direction.

ggg. Twist on one leg in place.

hhh. Same with the other leg.

5. Fifth beat:

A line of companions clapping and raising their legs alternately, while each group takes turns dancing and improvising their own steps.
